# Stand-Alone Sacroiliac-Joint Fusion as Novel Treatment Approach for Septic Arthritis of the Pubic Symphysis

**DOI:** 10.3390/medicina62020309

**Published:** 2026-02-02

**Authors:** Franz-Joseph Dally, Maria Antonia Rupp Pardos, Ali Darwich, Sascha Gravius, Michael Hackl, Steffen Heinrich Schulz, Frederic Bludau

**Affiliations:** 1 Orthopedic and Trauma Surgery Center, University Medical Center, 68167 Mannheim, Germany; arupp@rupali.de (M.A.R.P.); ali.darwich@umm.de (A.D.); sascha.gravius@umm.de (S.G.); michael.hackl@umm.de (M.H.); steffen.schulz@umm.de (S.H.S.); frederic.bludau@umm.de (F.B.); 2 Medical Faculty Mannheim, University Heidelberg, 68167 Mannheim, Germany

**Keywords:** septic arthritis of the symphysis, symphysitis, sacroiliac fusion, posterior stabilization, pelvic stability, posterior pelvic ring instability, sacral insufficiency fractures

## Abstract

Management of septic arthritis of the pubic symphysis (SAS) presents with substantial clinical challenges. Firstly, the SAS is an extremely rare entity. Surgical resection of the symphysis plus targeted antibiotic therapy is a widely adopted treatment course. Some patients suffering from SAS develop posterior pelvic insufficiency fractures because of the weakened anterior pelvic ring or as a result of radiation therapy received during treatment for a malignant disease in the lesser pelvis. The literature demonstrates a lack of standardized strategies for restoring pelvic ring integrity based on pelvic instability and posterior pelvic insufficiency fractures caused by SAS. *Background and Objectives*: This study aimed to determine whether early, primary stand-alone dorsal fusion can be a viable treatment option in SAS and whether there is a clinical benefit compared with temporary anterior fixation or secondary posterior stabilization after failed anterior fixation. *Materials and Methods*: We performed a descriptive, retrospective analysis covering an eight-year period (2018–2025) including 21 patients who underwent symphyseal resection for destructive SAS. We evaluated peri- and postsurgical data to describe the different surgical methods and their respective outcomes. *Results*: Ten patients (10/21, 48%) received posterior stabilization (sacroiliac-joint fusion or spinopelvic stabilization). Seven patients (7/21, 33%) were anteriorly fixated either temporarily with an external fixator or permanently with ventral plate osteosynthesis. Four patients (4/21, 19%) did not receive any pelvic stabilization following symphyseal resection as pelvic integrity was present. Three of them (3/21, 14%) showed spontaneous sacroiliac-joint fusion, while 6/7 (86%) of anteriorly fixed patients presented with debilitating sacral insufficiency fractures, had longer hospital stays and a higher count of readmissions and re-operations. Primary posterior stabilization led to shorter hospital stays, less readmissions, and good clinical outcome. *Conclusions*: Primary posterior stabilization can be a viable course of treatment of SAS and should be considered especially when spontaneous sacroiliac-joint fusion is not present. We suggest that early stabilization of the posterior pelvic ring could be a sensible course of treatment and may prevent debilitating insufficient fractures. While there are many different surgical options for posterior stabilization available (spinopelvic/lumbosacral stabilization, sacroiliac-joint fusion and others), our preliminary data suggest that primary sacroiliac-joint fusion is a quick, minimally invasive and effective way to establish posterior pelvic stability.

## 1. Introduction

Successful treatment of septic arthritis of the pubic symphysis (SAS) often requires debridement through wide resection of the pubic symphysis alongside antibiotic therapy [1,2,3,4,5], and in a number of cases, posterior pelvic stabilization of some kind due to sacral insufficiency fractures. As our previous study showed, symphyseal resection leads to heightened sheer forces on the posterior pelvic ring and increased risk and probability of sacral insufficiency fractures [6]. The pelvis is unique in its biomechanical architecture, functioning as a critical conduit for the transmission of forces from the upper body to the lower extremities. The posterior aspect of the pelvis bears the majority of load-induced stresses acting on the body [7]. In our previously assessed cohort, we showed that over the course of development and treatment of septic arthritis of the pubic symphysis (SAS), sacral insufficient fractures have a high incidence [6]. The development of insufficiency fractures of the sacrum in patients suffering from a septic arthritis of the pubic symphysis (SAS) is attributed to the compromised anterior pelvic integrity, pelvic radiation for treatment of a malignant disease in the lesser pelvis, and heightened sheer forces on the posterior pelvic ring.

Septic arthritis of the pubic symphysis is a very rare condition; however, it can be predominantly found in certain cohorts of patients, for instance, in patients who have had multiple diagnostical and surgical intervention in the small pelvis due to urological or gynecological cancers or other pathological processes occurring close to the pubic symphysis (PS). Moreover, the development of SAS is also linked to radiotherapy of the lesser pelvis [3], increasingly so after the treatment of urologic malignancies involving combinations of radical surgery and multiple radiation interventions [1,6]. Previous studies have shown that there can be a great latency from intervention or surgery in the lesser pelvis and the development of SAS, which in turn makes it difficult to detect and diagnose. In severe cases, symphyseal fistulas may form, most commonly between the bladder and symphysis but as stated, they also occur as rectal-/vaginal-/vesico-/prostato-symphyseal fistulas [1,2,3,4,5,6]. SAS can be a debilitating condition, especially when patients suffer from chronic infections and fistulas and lose anterior pelvic integrity, which, according to our previous study, can lead to insufficiency fractures of the sacrum in up to 30% of cases unless there is a spontaneous or preexisting fusion of the sacroiliac joints [6]. While other studies have shown that sacral insufficiency fractures do occur, management of these debilitating entities in the context of SAS has not been researched to this point.

Surgical planning and treatment must be interprofessional, and apart from orthopedic/trauma surgeons resecting the symphysis and connecting bony and ligamentous structures and stabilizing posteriorly or providing fixation of the anterior part of the pelvic ring, fistulas and compromised organs also regularly need surgical intervention or resection (from the urology, gynecology or general surgery department).

As described in our recent study, we propose standardized diagnostic and treatment steps for SAS best following the algorithm we introduced [6]. This algorithm includes checking for sacral insufficiency fractures via CT (computer tomography) or MRI (magnetic resonance imaging) scan of the pelvis. Interestingly while Hansen et al. reported on 6/26 (23%) of patients suffering from sacral insufficiency fractures and having trouble mobilizing at the six week follow-up, surgical or other treatment options besides crutches were not discussed [1]. Unlike Shu et al., who were only able to report on six patients altogether and stated that pelvic instability did not occur, we observed pelvic instability preoperatively and postoperatively [4]. Gupta et al. reported on 10 patients, and while for 8/10 (80%) wide resection of the symphysis and connecting bony and connective tissue has been described, this urological focused publication did not report any sacral insufficiency fractures while follow-up ranged from 4 to 28 months [5].

As stated, we have observed sacral insufficiency fractures in up to 30% of our treated SAS cases over the last eight years [6].

Our initial treatment protocol involved symphyseal resection, antibiotics, and anterior stabilization of the pelvic ring via anterior pelvic external fixator (APEF), and in one case, primary plate osteosynthesis focusing on anterior stability of the pelvic ring. Observing loosening, persistent pain, and sacral insufficiency fractures with these patients, we adapted our treatment protocol and began to stabilize the anterior and posterior by external and internal fixators (posterior spinopelvic stabilization or sacroiliac-joint fusion). While these anterior and posteriorly fixated patients performed better, they maintained the burden of APEF application and weight-bearing restrictions.

In several patients, we did not observe sacral insufficiency fractures. These were patients with a resection limited to symphyseal debridement and practically no bony resection, and interestingly, patients presenting with spontaneously fused sacroiliac joints.

Based on these findings, we formulated the hypothesis that primary stand-alone sacroiliac joint (SIJ) fusion, and thus posterior stabilization of the pelvis, could be a surgical approach of obtaining pelvic ring stability following symphyseal resection in the treatment of SAS.

Early primary dorsal fusion allows for pelvic stability while being minimally invasive and less of a burden than anterior pelvic external fixator (APEF). Patients developing septic arthritis of the pubic symphysis are commonly of advanced age, and optimizing quality of life should be prioritized.

The purpose of this paper is to spread the fact that SAS patients can develop sacral insufficiency fractures at high rates (30%) while patients with spontaneously fused sacroiliac joints do not develop sacral insufficiency fractures, and to share the evolution of our knowledge in the treatment of SAS and report on our preliminary results for patients having been treated with minimal invasive primary SIJ fusion as a stand-alone posterior fixation after wide symphyseal resection.

## 2. Materials and Methods

We conducted a retrospective analysis including 21 patients who underwent treatment for SAS at our institution between 2018 and 2025. Patients were included if they had undergone pubic symphysis resection for SAS at our university hospital during the specified study period. The debridement technique was standardized and consistently applied in all patients, involving resection of the superior and inferior pubic rami and the symphysis into healthy, non-infectious tissue by osteotomes, Luer, and Rongeurs. Wide resection was routinely performed with the removal of fistulas and infected organs such as the bladder if necessary. When indicated, drainage of abscess formations in the surrounding adductor muscle groups was additionally carried out.

A total of 21 patients were investigated in this study. We focused on surgical interventions to stabilize the pelvic ring. Widely accepted standard of care for most of the observation time was resection of the symphysis, in most cases accompanied with wide resection of parasymphyseal bone and connective tissues and anterior stabilization via APEF. Two years ago, we stopped APEF application and started posterior pelvic ring fixation alone using specialized 3D-printed titanium sacroiliac-joint fusion implants. As recently as a year ago, an updated version of the 3D-printed titanium fusion implant was released, and is now exclusively used for SIJ fusion.

Based on the course of pelvic stabilization, which varies according to clinical indications and presentations (loosening of implants, secondary sacral insufficiency fractures, and internal adaption of the treatment course over time), we identified four groups that the study cohort could be divided into:

1. Anterior pelvic fixation subgroups are represented by the Anterior Pelvic External Fixation (6/21) and Ventral Plate Osteosynthesis Cohorts (1/21), respectively (7/21, 30%).
2. Patients that received anterior pelvic fixation but developed debilitating sacral insufficiency fractures needing additional posterior stabilization; this is represented by the Dorsal Fusion Secondary to anterior pelvic fixation Cohort 3/21 (14%).
3. Patients receiving early, primary sacroiliac-joint fusion or posterior spinopelvic stabilization, this is represented by the Primary Dorsal Fusion Cohort 7/21 (30%)
4. Patients who did not need additional stabilization, represented by the No Stabilization after Symphyseal Resection Cohort 4/21 (19%).

The following parameters were evaluated in the present cohort: duration of surgery, mobility upon discharge or at first follow-up appointment, cumulative time of hospital stay (including cases where readmission was necessary for definitive stabilization/treatment due to instability or persistent discomfort), peri- and postoperative complication rate, readmissions after discharge as well as necessity for and type of reconstructive surgery.

The cumulative length of hospital stay—comprising all admissions required for successful treatment including readmission for definitive pelvic stabilization—was recorded starting from the date of symphyseal resection, rather than from the initial admission or referral.

For better clarity, the total duration of hospital stay was stratified into three groups: 5–20 days, 20–39 days, and over 40 days and evaluated.

The final study cohort (as Table 1 shows) consisted of four females (4/21, 19%) and seventeen males (17/21, 81%). The mean age at diagnosis of SAS was 73 years (+/−7.9 years standard deviation—SD). Nineteen patients had been treated for urological carcinomas (19/21, 91%) before developing septic arthritis of the symphysis. Urological entities observed were: carcinoma of prostate (16/21, 76%) and bladder carcinoma (3/21, 14%—including one case each of squamous cell carcinoma of the bladder, bladder adenoma, and urothelial carcinoma of the bladder).

One patient presented with a case of chronic sigma diverticulitis prior to development of SAS (1/21, 5%), while 95% of patients in this cohort (20/21) had radiation therapy at the level of the pelvis. All patients underwent resection of the pubic symphysis. Resection and debridement technique was standardized across all cases, and the methods of anterior and posterior stabilization varied, if applied. Seven patients received some kind of anterior fixation (7/21, 33%), either as temporary external anterior fixation or as ventral plate osteosynthesis. Six patients (6/7, 86%) received an anterior pelvic external fixator alone (mean duration: 41 days) following symphyseal resection. Ten patients (10/21, 48%) received dorsal stabilization as part of adequate SAS treatment; in three cases, this was preceded by temporary external fixation (3/10, 30%). One patient received dorsal fusion before symphyseal debridement due to insufficiency fracture of the sacrum and diagnostic delay of SAS because he was admitted to another hospital; this patient did not receive external anterior fixation at any point. Seven patients (7/10, 70%) underwent symphyseal resection and dorsal fusion without subsequent anterior external fixation.

Four patients (4/21, 19%) did not receive any kind of temporary or permanent stabilization following resection. Three of these four patients (3/4, 75%) showed idiopathic sacroiliac fusion and there was no need for additional stabilizing surgery; the remaining patient did not classify for stabilizing surgery due to the minimal extent of resection in SAS treatment.

## 3. Results

### 3.1. Pain Levels

All patients had high pain levels before symphyseal debridement and surgical intervention (pre-operative VAS—Visual Analog Pain Scale 7.6/10; +/−2.7 SD). Pain was described as sharp and mostly located in the groin and the anterior pelvic ring/symphyseal region and worsened during gait, ambulation, or weight bearing. Postoperatively, a few distinctions need to be made. Initially, the anterior or symphyseal pain increased sharply for all patients to 5.4/10; +/−1.8 SD, which can be attributed to the debridement of infected tissue and abscess drainage.

The APEF cohort (6/21; 29%) reported higher pain levels and discomfort during the external fixator application period. In particular, sitting was an issue, since the external fixator caused pain during hip flexion. A total of 30% of the APEF patients over time developed sacral insufficiency fractures and in turn lost the ability to ambulate, and the VAS levels again increased sharply to 8.3/10; +/−0.7. These patients received secondary posterior stabilization surgeries, and 1/3 (33%) needed numerous surgeries.

The primary dorsal fusion cohort (7/21; 33%) showed the same decrease in pain levels to 4.7/10; +/−1.9 SD regarding the anterior pelvic pain, while 6/7 (86%) of the dorsal fusion group reported maintained low pain levels postoperatively at six weeks follow-up (4.5/10; +/−2.1 SD). One patient showed loosening of his SIJ fusion implants, lost the ability to ambulate due to worsening pain, and needed lumbopelvic revision surgery, after which his pain levels improved and he was able to ambulate with a walker.

A total of 4/21 (19%) did not need surgical interventions besides symphyseal resection, and their pain levels decreased as effectively as for the other patients, while 3/4 (75%) of those patients received wide resections of the symphysis and adjacent bone and connective tissue, and one patient received a sole debridement of the symphyseal cartilage via Luer and Rongeurs.

None of the patients with wide resections showed signs of sacral insufficiency fractures although they had received the same treatment for a malignant entity in the lesser pelvis (chemotherapy and radiation).

We further examined the patients who seemed resistant to pelvic ring instability or sacral insufficiency fractures and noticed key findings: they all presented with spontaneous SIJ fusions. Every patient who developed sacral insufficiency fractures in the context of SAS treatment, on the other hand, did not present with spontaneous SIJ fusion.

### 3.2. Radiological Signs in Patients with Spontaneous Idiopathic Sacroiliac Fusion

Three of the four patients who did not receive any additional stabilization of the pelvic ring showed spontaneous sacroiliac fusion. These patients did not suffer from sacral insufficiency fractures.

**Figure 1:**
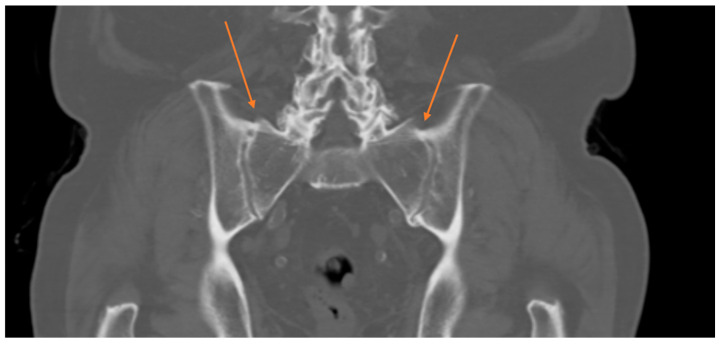
Coronal CT of the pelvis showing signs of spontaneous sacroiliac-joint fusion cranial and anterior of the SI joint (arrows) making posterior stabilization of the pelvis unnecessary despite wide resection of the symphyseal region.

**Figure 2:**
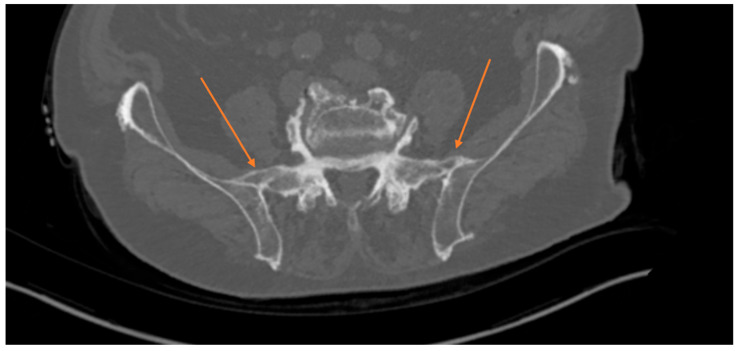
Axial CT of the pelvis showing signs of spontaneous sacroiliac-joint fusion cranial and anterior of the SI joint (arrows) making posterior stabilization of the pelvis unnecessary despite wide resection of the symphyseal region.

Radiological signs observed in patients with spontaneous sacroiliac fusion were:

Osseus bridges across the sacroiliac joints, which can be found cranially, anteriorly, posteriorly or within the joint (Figure 1 and Figure 2).

Sacral and iliac cortices connected by bony formations anteriorly (most common area of idiopathic fusion), cranially, or within the joints (the latter being the rarest form of idiopathic fusion).

### 3.3. Mobility

Mobility was evaluated preoperatively, at time of discharge, and at the first clinical follow-up 6 weeks after surgery. Patients were either mobile with aid, (forearm crutches, walker) mobile unaided (able to walk freely without any aid), or immobile but able to stand.

Preoperatively, across all groups, symphyseal pain was high and prevented patients from walking far. The highest mobility levels besides symphyseal pain were reported by the patients with spontaneous SIJ fusion as they were able to ambulate roughly 500 m by their own estimation with the aid of a walker. The worst ambulatory levels were observed when patients developed sacral insufficiency fractures. Because of the radiation therapy combined with anterior pelvic instability after wide bony resection, these patients (7/21; 33%) showed no sign of spontaneous healing and presented to our clinic bedridden.

Mobility at 6 week follow-up:

- Anterior Fixation Cohort (n = 7):
  ○ Anterior External Fixation:
    - Mobile with the use of aid: 5/6 (83%);
    - Immobile but able to stand: 1/6 (17%).
  ○ Ventral Plate Osteosynthesis:
    - Mobile with the use of aid (n = 1).
- Dorsal Fusion Cohort (N = 10):
  ○ Dorsal Fusion Secondary to Anterior Pelvic External Fixation (N = 3):
    - Mobile with the use of aid: 1/3 (33%);
    - **Mobile unaided: 2/3 (67%).**
  ○ Primary Dorsal Fusion (n = 7):
    - Mobile with the use of aid: 4/7 (57%);
    - **Mobile unaided: 3/7 (43%).**
- No Stabilization after Symphyseal Resection (n = 4):
    - Mobile with the use of aid: 2/4 (50%);
    - **Mobile unaided: 2/4 (50%).**

Overall, there was no significant difference between these groups. Patients with a stable posterior stabilization, either primary or secondary or via spontaneous SIJ fusion, were the only groups where patients were able to ambulate unaided at six weeks follow-up. In the APEF group, mobility was further impaired and lower than for the other cohorts with a stabile posterior pelvic ring. Again, while there were no levels of significance to report, we observed a tendency toward a better outcome and mobility when the posterior pelvic ring was stabilized.

### 3.4. Cumulative Time of Hospital Stay

First, we present an overview of the length of stay for the entire cohort which will be revisited in Table 2. Ten patients spent 5–20 days in hospital after symphyseal resection (10/21, 48%); three patients spent 20–39 days (3/21, 14%); and the remaining eight patients spent more than 40 days (8/21, 38%).

- Anterior Fixation:
  ○ Anterior Pelvic External Fixation:
    - 5–20 days: 1/6 (17%);
    - 20–29 days: 3/6 (50%);
    - >40 days: 2/6 (33%).

**Mean duration of hospital in this subgroup: 34.2 days (+/−9.2 SD).**

  ○ Ventral Plate Osteosynthesis:
  The patient who received a tricortical block and symphyseal screw and ventral plate osteosynthesis stayed in the hospital for 42 days for SAS treatment.

- Dorsal Fusion Secondary to External Fixation:
  ○ 5–20 days: 1/3 (33%);
  ○ >40 days: 2/3 (67%).

**Mean duration: 62 days (+/−60 SD).**

- Primary Dorsal Fusion:
  ○ 5–20 days: 6/7 (86%);
  ○ >40 days: 1/7 (14%).

**Mean duration: 21.3 days (+/−16 SD).**

- No Stabilization after Symphyseal Resection:
  ○ 5–20 days: 2/4 (50%);
  ○ >40 days: 2/4 (50%).

**Mean duration: 39 days (+/−30.8 SD).**

Regarding hospital stay, while no significant levels were reported, patients with primary dorsal fusion had the shortest hospital stay.

The longest duration spent in hospital were patients who showed sacral insufficiency fractures after having received APEF application. These patients received, at times, complex spinopelvic stabilization surgeries. When posterior stabilization was conducted after sacral insufficiency fractures occurred, the mean duration of hospital stay was three times as long as compared to the primary dorsal fusion cohort. This a glaring discrepancy.

**Necessity of Readmission:**
  - Anterior Fixation:
    ○ Anterior External Fixation: 3/6 patients (50%) needed to be readmissioned.
    ○ Ventral Plate Osteosynthesis: The ventral plate patient needed to be readmissioned to hospital for further treatment.
  - Dorsal Fusion Secondary to External Fixation: **2/3 (67%) patients needed to be readmissioned.**
  - Primary Dorsal Fusion: **1/7 (14%) patients needed to be readmissioned.**
  - No Stabilization after Symphyseal Resection: No patients in this cohort needed to be readmissioned for SAS treatment.

Only 1/7 (14%) of patients receiving primary dorsal fusion needed to be readmissioned. We observed that 67% of the patients with secondary posterior stabilization needed to be readmissioned. This was for further surgical intervention and revision because of surgical site infections after spinopelvic surgeries.

### 3.5. Number and Duration of Surgical Procedures

Among the 21 patients analyzed in this subgroup, a total of 51 surgical procedures were performed to address septic arthritis of the symphysis. Surgical interventions included symphyseal resection, cystectomy (or prostate/vesiculectomy), fistula excision, and pelvic ring stabilization. Cystectomy and prostate/vesiculectomy procedures were included in the analysis only when performed within the same operative session as an intervention for the treatment of SAS and associated pathologies such as symphyseal fistulas.

The mean number of surgical interventions per patient was 2.43.

The cumulative duration of surgical interventions was assessed, recognizing variation in the number of procedures required to achieve definitive management of the underlying disease. Depending on case complexity, some patients underwent a single operation, while others required multiple consecutive procedures to achieve favorable clinical outcome. The total operative time—mean: 355 min, range 142–529 min—represents the sum of all surgical interventions performed for the treatment of SAS over the eight-year study period, either conducted as a single surgery or as successive procedures.

**Figure 3:**
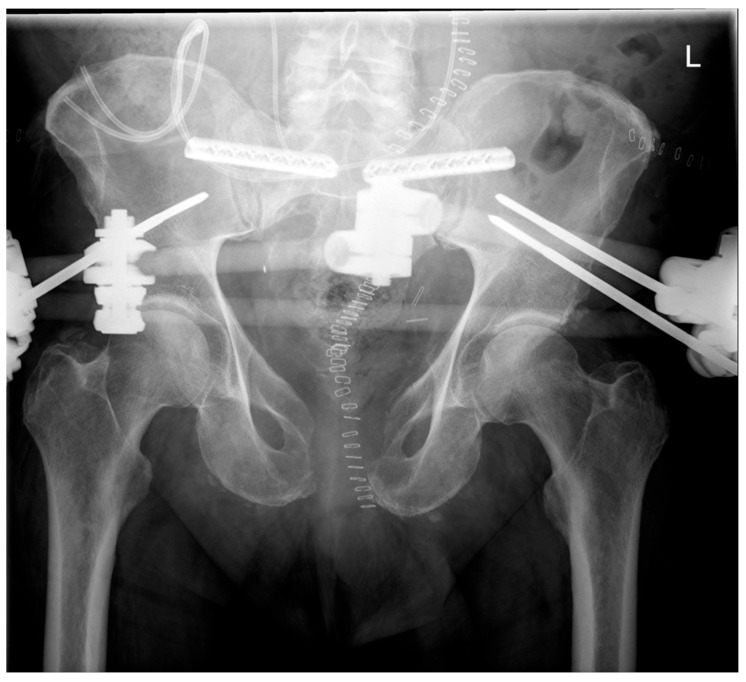
iFuse^®^ implants to achieve dorsal fusion via SI-joint fusion shown in an X-ray of the pelvis, a.p., resected symphysis anterior, anterior pelvic external fixator implanted, overly of catheters from ileostomy, and pig tail double J catheters into the kidneys.

**Figure 4:**
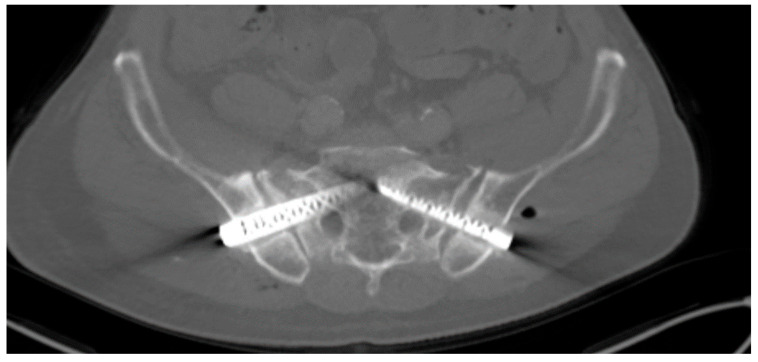
iFuse^®^ implants showing signs of loosening in an axial CT scan prior to removal in a patient who had received delayed SI-joint fusion when insufficiency fractures caused by osteonecrosis had already formed.

**Figure 5:**
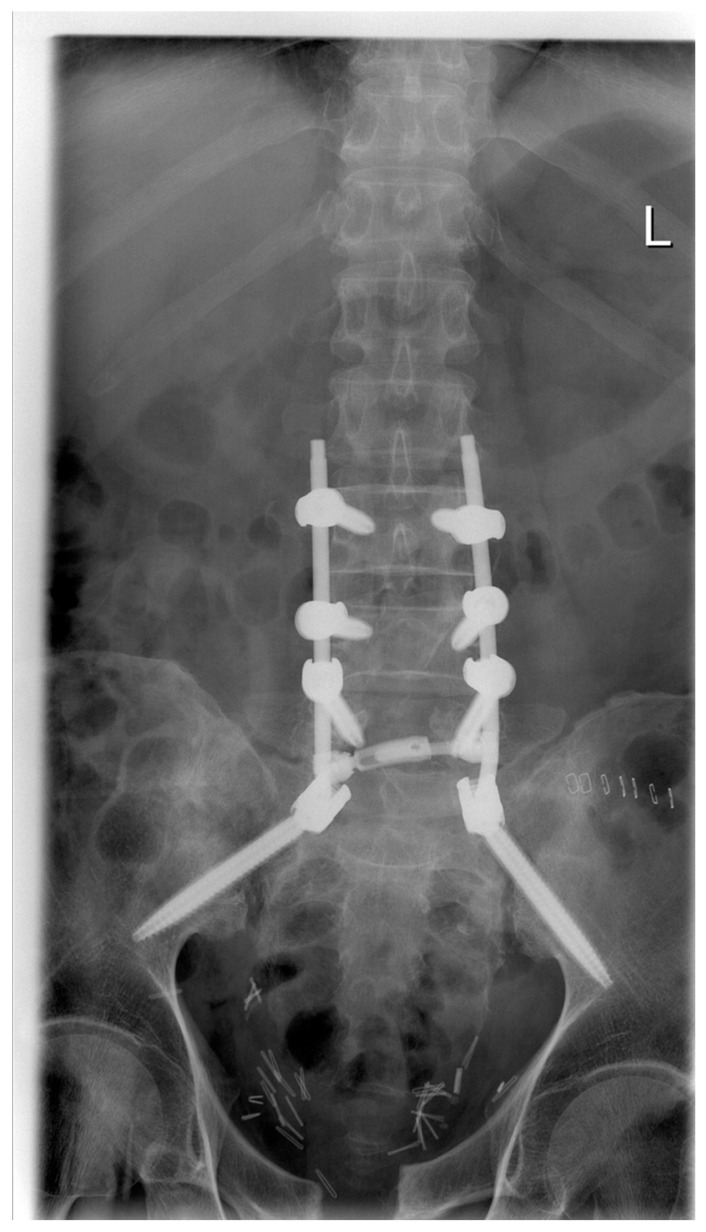
Showing the same patient as in Figure 4 in a coronal standing X-ray following loosening of implants, removal, and restabilization via spinopelvic stabilization (Stryker, K2M, EVEREST^®^) L3–S2–Ala–Ilium.

- Anterior Fixation:
  ○ Anterior External Fixation: Mean: 314 min (range 142–529 min);
  ○ Ventral Plate Osteosynthesis: 482 min (n = 1).
- Dorsal Fusion Secondary to External Fixation (Figure 3, Figure 4 and Figure 5):

Mean: 380 min (range 239–505 min).

- Primary Dorsal Fusion:

Mean: 406 min (range 320 min).

- No Stabilization after Symphyseal Resection:

Mean: 236 min (range 120–373 min).

### 3.6. Necessity of Revision Surgeries Beside Posterior Pelvic Revision

- Anterior Fixation:
  ○ Anterior External Fixation: 3/6 patients (50%) needed reconstructive surgery in SAS treatment, procedures performed were: two patients needed a pedicled greater omentum flap performed during urological part of the interdisciplinary surgery (urological), and one patient needed a rectus abdominis muscle flap for soft tissue coverage (orthopedic).
  ○ Ventral Plate Osteosynthesis: No reconstructive surgery was necessary in this case.
- Dorsal Fusion Secondary to External Fixation: One patient in this cohort (1/3; 33%) needed reconstructive surgery, where a rectus abdominis muscle flap was needed for soft tissue coverage (orthopedic).
- Primary Dorsal Fusion: Two patients (2/7; 29%) needed reconstruction, one of the abdominal wall and peritoneum as part of the interdisciplinary surgery (urological), and one abdominal fascia closure in fascia dehiscence. Regarding the posterior stabilization, there was no need for revision surgery.
- No Stabilization after Symphyseal Resection: One patient in this cohort needed a pedicled greater omentum flap for persistent fluid formation in the lesser bowel and defect coverage (urological aspect of surgery) (1/4; 25%).

### 3.7. Clinical Outcome

Satisfactory outcome was measured as changes on the Oswestry Disability Index (ODI) scale. We performed ODI at 6 weeks follow-up after the last surgical intervention; where available, the ODI was calculated via follow-up documentation that readily included questions to walking distance, pain, selfcare, walking, lifting, and so on.

### 3.8. Anterior Fixation

A total of 2/6 (33%) patients who received an APEF demonstrated satisfactory clinical outcome (mobility, pain levels, pelvic stability), but almost all patients (5/6; 83%) needed multiple surgical procedures to eventually reach that level. Their ODI levels ended up at 30.

Four out of six (67%) APEF patients demonstrated an unsatisfactory clinical outcome. One patient developed septic shock, and one patient developed sacral pseudarthrosis after insufficiency fracture, constituting persistent severe pelvic ring instability. Pain management and therapy were escalated and successful, as no further surgeries were agreed upon by the patient.

Two patients suffered from prolonged hospital stays and readmissions for pelvic instability, wound complications, and further surgical procedures. Their ODI levels ended at 60 +/− 9.

One patient received, in an off-label surgery, a symphyseal debridement and ventral plate osteosynthesisi and showed satisfactory clinical outcome (n = 1) but needed two surgical revisions for infected seroma. His ODI at six weeks was 40.

### 3.9. Dorsal Fusion Secondary to External Fixation

One out of three (33%) patients demonstrated satisfactory clinical outcome (mobility, pain levels, pelvic stability) with an ODI level of 36.

Two out of three (67%) patients demonstrated unsatisfactory clinical outcome. One patient presented with chronic low back pain due to right-sided pseudarthrosis of the sacrum, with pain adequately compensated; patient remained able to stand and ambulate short distances. One patient presented with chronic wound-healing complications resulting in further surgeries and persistent pelvic instability. Their ODI was 63 +/− 4.

### 3.10. Primary Dorsal Fusion

All patients in this cohort (7/7, 100%) eventually showed satisfactory clinical outcome. To achieve satisfactory clinical outcome in one patient, SIJ fusion implant removal and subsequent spinopelvic stabilization were necessary due to persistent instability and loosening likely related to poor bone quality (radioosteonecrosis). Their combined ODI was 31 +/− 12.

### 3.11. No Stabilization After Symphyseal Resection

All patients in this cohort (4/4; 100%) demonstrated satisfactory clinical outcome. Their ODI was 24 +/− 7.

### 3.12. Recently Implemented Surgical Approach

**Figure 6:**
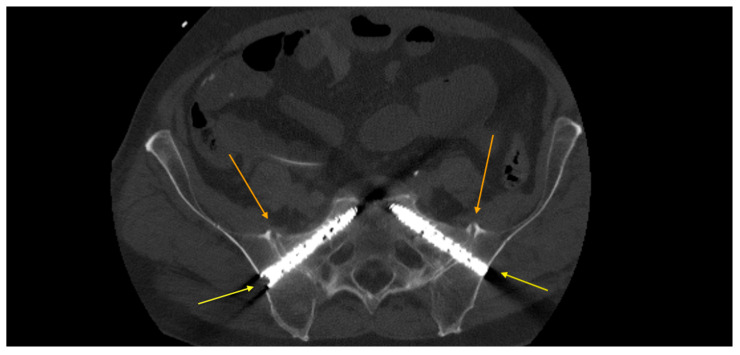
Sacroiliac-joint fusion via iFuse-TORQ implants^®^ shown in the CT-scan, axial view, with implants laying safely and entirely in the S1 corridor. Entry point is far posterior (yellow arrow) aiming ventrally toward the promontory as it is the area of the strongest bone formation. Notice the non-fused SI joints (orange arrows).

**Figure 7:**
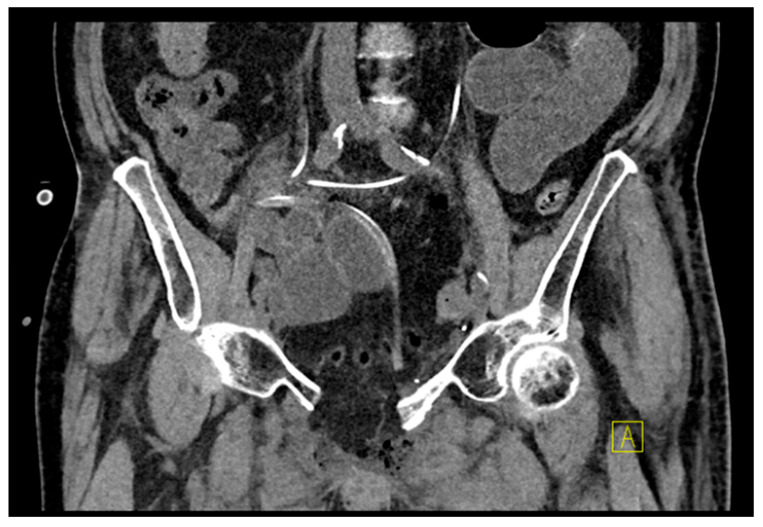
CT scan showing the resected symphyseal area with bony defect and multiple double J or ileostomy catheters after cystectomy for vesico-symphyseal fistula and chronic SAS.

Based on our observations over the last 8 years, we have changed our surgical approach to a “one-stop-surgical approach”:

1. Checking patient CT scans for signs of spontaneous SIJ fusion; if there is spontaneous fusion, no posterior surgery is performed, if there are no signs of SIJ fusion, we precede to the next step.
2. Stabilization of the dorsal pelvic ring via sacroiliac-joint fusion constitutes the first step of the multidisciplinary surgical procedure and is performed with the patient in the prone position (Figure 1). We advise using whichever technique the surgeon is most comfortable with to stabilize the posterior pelvic ring. We chose SIJ fusion implants as they manage to primarily stabilize the posterior pelvic ring and induce an SIJ fusion (Figure 6)
3. Following dorsal fusion of the SIJ, the patient is repositioned supine for the anterior approach, during which the symphysis, infected pubic bone, and infected surrounding connective tissue is removed (Figure 7). After bony and tissue resection and debridement by the orthopedic/trauma team, a surgical team of the urological department continues with cystectomy, fistula resection, and ileostomy for urine secretion, if needed, paired with other surgical teams (general surgery, gynecology, etc.).

A total of 7/21 (33%) patients in this study were operated on via our new approach using sacroiliac-joint fusion devices.

As our results show, posterior stabilization of SAS patients leads to shorter days in the hospital and lesser percentages of readmissions, while eventually showing a satisfactory result according to ODI levels.

One finding that was extremely interesting is how patients with spontaneous SIJ fusion have very good results while seemingly having a very low to non-existent risk for sacral insufficiency fractures even after radiation and wide resection of the symphyseal region.

## 4. Discussion

The clinical presentation of septic arthritis of the pubic symphysis (SAS) varies widely due to the heterogeneity of the infectious process, which may lead to progressive destruction of the symphysis and adjacent soft tissues, extension into the adductor musculature as well as formation of fistulous tracts to neighboring organs—most commonly between the pubic symphysis and the bladder. Diagnosing this debilitating condition remains challenging, and no standardized diagnostic nor surgical or non-surgical treatment approaches have been established to date. Based on these findings, we proposed a diagnostic and therapeutic algorithm [6].

Over the course of the last eight years, we have gained valuable knowledge into this complex pathological entity—septic arthritis of the symphysis caused by treatment of a malignant entity in the lesser pelvis. Mostly, this cohort is predominantly composed of patients who have undergone treatment for urologic malignancies, which is in line with the other literature [1].

In our clinical observation, at least 30% of the patients suffered from sacral insufficiency fractures at some point after SAS treatment, which made revision surgeries and, at times, long posterior stabilization constructs necessary [1,2,3,4,5]. Most of the literature on this topic so far originates from one urologic study group, making the lack of focus on sacral insufficiency fractures as a concomitant pathologic entity less surprising [2,3,5].

We further observed that patients treated with an anterior external fixator reported markedly reduced quality of life, as seen in their ODI values, whereas those undergoing posterior fixation and stabilization experienced less pain, better comfort, and comparable mobility, without the morbidity associated with external fixation, which is mirrored in their ODI values. The anterior stabilization patients eventually reported satisfying levels of mobility, but 5/6 (83%) had to undergo further surgeries, in some cases needing major plastic reconstructive surgeries.

In our study, interestingly, patients (3/21; 14%) had wide resections of the symphyseal region and their surrounding bony and connective tissues without any sacral insufficiency fractures. The reason for not developing fractures seemingly was the presence of spontaneous fusion of their SI joints. The number of hospital days were lower, and their ODI values were better than for the APEF group or for the cohort with secondary posterior stabilization.

Based on our findings of patients with SIJ fusion not developing sacral insufficiency fractures and primary posterior stabilized patients doing better, we changed our treatment regimen from symphyseal resection and APEF application to a “one-stop-surgical-approach”, stabilizing the posterior as the first surgical treatment step.

While lumbopelvic posterior stabilization had to be performed in 4/21 (19%) patients, there is a known morbidity (infections, revision surgery, loosening of sacroiliac screws) attached to this surgical option. We therefore chose percutaneous sacroiliac-joint fusion as our posterior stabilization option.

In our clinical practice, percutaneous sacroiliac fusion is a minimally invasive procedure and is now employed in patients with symphyseal resection due to SAS to reduce the worsening [8] and possible occurrence of insufficiency fractures that are associated with higher morbidity and mortality in patients [9].

Reduced morbidity, immediate primary stability, and realization of secondary lasting posterior pelvic ring stability (in line with patients with a spontaneously fused SIJ), highlights the potential advantages of immediate percutaneous sacroiliac fusion following symphyseal resection.

The aim of this study was to evaluate stand-alone dorsal fusion as a pelvic stabilization method in complex cases of septic arthritis of the symphysis.

Treatment of SAS is complex and challenging, which is reflected in the duration of surgical interventions, with a mean operative time of 355 min (range 142–529 min), number of readmissions, and number or reoperations.

The SAS cohort is a potentially fragile one. As our cohort reflects, a typical SAS patient has had treatment for a malignant entity in the lesser pelvis, where a large number of them deal with debilitating fistulas (most commonly between bladder and symphysis but also rectal-/vaginal-/vesico-/prostato-symphyseal) and persistent infections that can be difficult to detect in the first place [6]. Treatment focus for these patients must be to limit the surgical time exposure as well as limit the number of surgeries.

Until two years ago, at our university, clinical procedures were not standardly performed during a single interprofessional surgical session but were at times divided into an orthopedic and urologic, general surgery, or gynecology surgery.

Our new surgical approach is, as stated before, now performed as a “one-stop-surgical-approach”, underscoring the shift toward early posterior stabilization and the multidisciplinary approach to SAS treatment and focusing on early definite treatment for this fragile patient cohort by performing all necessary surgeries in one interprofessional setting.

In our cohort, the four patients who did not receive stabilization after symphyseal resection showed good clinical function at discharge, suggesting that intact pelvic integrity based on spontaneous or preexisting fusion of the sacroiliac joints may allow for the omission of fixation.

Indeed, we were able to radiologically confirm idiopathic sacroiliac fusion in three of these four patients. The patient who did not show sacroiliac-joint fusion had a very limited extent of anterior symphyseal resection, therefore further strengthening the posterior pelvic ring integrity was not necessary.

Notably, we would like to highlight the cases where idiopathic fusion of the posterior pelvic ring occurred, resulting in satisfactory pelvic integrity without surgical stabilization.

In treatment of SAS patients and planning surgical resections of the symphysis and surrounding bony and connective tissue aspects, we now regularly check for SIJ fusion and if apparent, do not see reason to stabilize these patients posteriorly. We hypothesize that in other publications, while not being noted, the spontaneous fusion of SI joints might have been apparent, as well above 70% of the patients were male and elderly, possibly living with ankylosing spondylitis, and thus fusion of their SI joints [3,5].

Radiological signs observed in patients with spontaneous sacroiliac fusion that should be routinely examined for, are:

Osseous bridges across the sacroiliac joints and sacral and iliac cortices connected by bony formations anteriorly, cranially, or within the joints.

This observation further supports the notion that dorsal fusion alone can provide sufficient pelvic stability without the need for APEF. Moreover, achieving such fusion through minimally invasive techniques such as percutaneous procedures may offer optimal clinical outcomes while reducing treatment-related burden.

The treatment concept of using a temporary anterior pelvic external fixator is based on waiting for and supporting fibrous healing, which may occur in some patients, resulting in a more rigid anterior pelvic ring providing pelvic ring stability even without additional permanent anterior or posterior stabilization. This describes the underlying process taking place in a few patients presenting with effective wound healing and non-compromised wound-healing. However, as most patients in our SAS cohort (20/21; 95%) had undergone radiation of the lesser pelvis, anatomical structures having undergone radiation therapy are more susceptible to worsened wound healing and regenerative issues [10]. In vitro studies show that irradiated osteoblasts and fibroblasts exhibit a prolonged and dose-dependent reduction in collagen production compared to controls [11]. Furthermore, osteoblast and fibroblast proliferation is inhibited after radiation, and slower growth rate is mainly attributed to reduced proliferation activity rather than initial cell death caused by radiation [11]. In patients with intensive radiation history or multiple surgeries, tissue and bone healing is often compromised, and sacral insufficiency fractures are more likely. All of these known side-effects of radiation therapy are observed in SAS patients on the grounds of cancer treatments, fistula formations, and chronic infections [12]. Our cohort demonstrated concurrently how no fibrous healing or tight pseudarthrosis formed in the bony symphyseal resection defect, and spontaneous stabilization of the pelvic ring does not seem to occur in patients with wide symphyseal resections.

The patient cohort with an SAS is a vulnerable one, and overall disease burden associated with SAS is considerable. Therefore, minimizing the hospital length of stay (LOS) and avoiding readmissions are essential to prevent further disease burden.

In our cohort, the shortest mean hospital stay was observed in patients who underwent primary dorsal fusion, averaging 21.3 days, with only one patient (14%) requiring readmission for SAS-related treatment. In contrast, among patients who did not receive additional stabilization, none required readmission; however, the mean hospital stay in this group was notably longer at 39 days. Three times as high (63 days) was the mean LOS times in patients who received a secondary posterior stabilization after failed APEF/anterior stabilization.

Considering the lower readmission rate, shorter length of hospital stay, better ODI values, and equal mobility of our sample cohort of early, primary surgically sacroiliac fused patients, we believe that routine posterior stabilization should be standard of care for SAS treatment in which symphyseal resection is performed.

This is justified to prevent subsequent and debilitating sacral insufficiency fractures.

Our case and thinking are strengthened by the positive outcome of patients with spontaneously fused sacroiliac joints.

The final choice of the surgical approach appears to depend on multiple patient-specific factors including previous interventions, bone quality, and the operating surgeon’s experience.

According to our observations, we continue our recently implemented surgical approach. The data suggest that SI-joint fusion methods should be prioritized to enhance pelvic ring stability, as they improve clinical outcomes while reducing morbidity.

Acknowledging the heterogeneity of our cohort and the various treatment approaches throughout the eight-year study period, we wish to highlight the complexity of SAS treatment and the need for experienced professionals in finding the adequate treatment. Patients should be treated at an institution where various specialists for SAS treatment are readily available (orthopedics/trauma surgery, reconstructive surgery, urology, gynecology, general surgery, and microbiology).

This article aims to share our insights and the evolution of our clinical expertise. We have found sacroiliac fusion to be a promising approach in SAS treatment. The pelvic integrity and stability seen in cases where idiopathic sacroiliac fusion occurred naturally provided sufficient pelvic stability, protecting the individuals from sacral insufficiency factures. While we have not gathered long-term outcome data for this new surgical approach at our institution, we have shown that posterior stabilization should be the goal, whether this is realized by lumbopelvic stabilization or minimally invasive by percutaneous SIJ fusion or, for instance, any other posterior pelvic treatment option such as transsacral bar osteosynthesis, was not focus of this study.

Due to the retrospective study design, comparison between surgical approaches was limited.

Further studies are needed to strengthen our hypothesis and provide further insights in the optimal treatment for SAS patients.

## 5. Conclusions

This study is, to the best of our knowledge, the first to present the need and compare surgical methods of pelvic ring stabilization in symphysectomy patients for the treatment of septic arthritis of the pubic symphysis (SAS) for developing sacral insufficiency fractures.

The development of sacral insufficiency fractures in SAS patients to this point has been glaringly underreported. We observed that at least 30% of these patients will develop debilitating fractures that will further complicate their treatment and limit their outcome.

We showed that spontaneous sacroiliac-joint fusion appears to be a protective manifestation as it was associated with preserved mobility, reduced morbidity, lesser LOS compared, and good ODI values.

We implore everyone involved in the treatment of SAS to look for the spontaneous fusion of SI joints and report on it as it, in our observation, changes the surgical treatment course fundamentally as these patients do not need posterior stabilization. We have in this study, for the first time, provided concrete pointers that can be followed to determine whether SIJ fusion is apparent. When SIJ fusion is not certain, we advise posterior stabilization.

We were able to show that primary posterior stabilization is a superior treatment option compared to anterior stabilization.

Percutaneous SIJ fusion is our surgery of choice for dorsal fusion as it is minimally invasive, needing little to no readmissions and barely requiring revision surgeries.

Patients treated via our “one-stop-surgical-approach” have so far been performing better than APEF or any other surgery subgroup in this study.

Based on our findings, we recommend dorsal fusion via sacroiliac-joint fusion in cases of SAS, particularly when extensive destruction of the symphysis is present and wide resection is necessary to provide infection control. While the results of this retrospective descriptive analysis are correlational, they highlight the potential advantages of sacroiliac-joint fusion as a minimal invasive approach of improving pelvic ring stability in SAS patients, warranting further prospective evaluation.

### Limitations and Outlook

This study is a retrospective descriptive analysis, and the findings primarily suggest a correlation rather than causality with regard to the best surgical care and clinical outcomes, highlighting the need for further prospective research to determine the optimal fixation strategy balancing infection control, biomechanical stability, and quality of life in the surgical treatment of septic arthritis of the pubic symphysis.

## Figures and Tables

**Table 1:** Characteristic of the study cohort.

Parameter	n (%)
SAS	21 (100%)
Mean Age at Diagnosis	73
Males	17 (81%)
Females	4 (19%)
Urological CA prior	19 (91%)
Prostate CA	16 (76%)
Bladder CA	3 (14%)
Radiation of Pelvis prior to SAS	20 (95%)
Surgical Stabilization	17 (81%)
No Surgical Stabilization	4 (19%)
Spontaneous SI-Joint Fusion	3 (14%)
APEF	6 (29%)
Ventral plate osteosynthesis	1 (5%)
Dorsal fusion	10 (48%)
2nd DF	3 (14%)
1st DF	7 (33%)

Table 1 shows the characteristics of the study cohort, n = 21. Abbreviations: SAS = septic arthritis of the pubic symphysis, APEF = anterior pelvic external fixation; DF = dorsal fusion; 2nd DF = dorsal fusion secondary to anterior external fixation; 1st DF = primary dorsal fusion; CA = carcinoma.

**Table 2:** Table of the analyzed parameters in the present cohort regarding surgical care, peri- and post-surgical parameters in the treatment of septic arthritis of the symphysis.

Parameter	APEF	VP	2nd DF	1st DF	NS
n = x	n = 6	n = 1	n = 3	n = 7	n = 4
Cumulative hospital stay (mean)	34.2 days	42 days (n = 1)	62 days	21.3 days	39 days
Necessity of readmission	3/6, 50%	1/1, 100%	2/3, 67%	1/7, 14%	0/4
Necessity of revision surgery	5/6, 83%	1/1, 100%	1/3, 33.3%	2/7, 29%	1/4, 25%

Subgroup analysis regarding different stabilization methods applied (if applied) in the treatment of septic arthritis of the symphysis, analyzed subgroups: APEF = anterior pelvic external fixation; VP = ventral plate osteosynthesis; 2nd DF = secondary dorsal fusion following external fixation; 1st DF = primary dorsal fusion; NS = no stabilization following symphyseal resection.

## Data Availability

Data used in this study were obtained from patient files and surgery reports archived at University Hospital Mannheim, Germany using the EPOS/SAP software. Radiological data were obtained through SYNGO software commonly used by the University Hospital Mannheim, Germany.

## Abbreviations

The following abbreviations are used in this manuscript:

SAS	Septic arthritis of the symphysis
SIJ	Sacroiliac joint
APEF	Anterior pelvic external fixation
CA	Carcinoma
VP	Ventral plate
DF	Dorsal fusion
1st DF	Primary dorsal fusion
2nd DF	Dorsal fusion secondary to external fixation
NS	No stabilization following symphyseal resection
CT	Computer tomography
